# Network meta‐analysis of novel and conventional sentinel lymph node biopsy techniques in breast cancer

**DOI:** 10.1002/bjs5.50157

**Published:** 2019-03-25

**Authors:** C. W. Mok, S.‐M. Tan, Q. Zheng, L. Shi

**Affiliations:** ^1^ Division of Breast Surgery, Department of Surgery Changi General Hospital Singapore; ^2^ Singapore Clinical Research Institute Singapore

## Abstract

**Background:**

The aim of this network meta‐analysis was to compare the performance of blue dye alone or in combination with radioisotope (technetium‐99m, Tc) with three novel techniques for sentinel lymph node detection in breast cancer: indocyanine green fluorescence (ICG), superparamagnetic iron oxide (SPIO) nanoparticles and contrast‐enhanced ultrasound imaging (CEUS).

**Methods:**

PubMed, Embase, the Cochrane Library, China Knowledge Research Integrated Database, ClinicalTrials.gov and OpenGrey databases were searched up to 31 November 2017, without language restriction. Studies that compared the detection performance of at least one of the novel methods (ICG, SPIO and CEUS) with that of traditional methods (blue dye and/or radioisotope) were included in network meta‐analysis.

**Results:**

Thirty‐five studies were included. Pooled risk ratios (RRs) for Tc (1·09, 95 per cent c.i. 1·04 to 1·15), ICG (1·12, 1·07 to 1·16) and SPIO (1·09, 1·01 to 1·18) showed statistically better performance in detecting sentinel lymph nodes than blue dye alone. ICG had the lowest false‐negative rate, with a RR of 0·29 (0·16 to 0·54), followed by Tc (RR 0·44, 0·20 to 0·96) and SPIO (RR 0·45, 0·14 to 1·45), with blue dye alone as the reference group.

**Conclusion:**

SPIO or ICG alone are superior to blue dye alone and comparable to the standard dual‐modality technique of blue dye with Tc.

## Introduction

Lymphatic staging of breast cancer was initially established by axillary lymph node dissection followed by sentinel lymph node biopsy^1^. There may be more than one sentinel node, depending on the location of the tumour and the network of surrounding lymph vessels^2^. For patients with early breast cancer and clinical and radiological absence of axillary lymph node metastases, sentinel lymph node biopsy is currently considered the standard of care^3^.

The most widely used technique for sentinel lymph node identification is the dual‐modality method involving the injection of technetium‐99m (Tc)‐labelled nanocolloid and blue dye (BD) into the peritumoral or periareolar region^4^. ‘Hot’ nodes detected using a handheld scintillation counter (γ‐probe) and ‘blue’ nodes identified during surgery are both recognized as sentinel nodes and removed, followed by histopathological confirmation of metastases. An identification rate of 96 per cent was reported in a large meta‐analysis^5^ of 8000 patients. In another meta‐analysis^6^ of more than 9000 patients, the false‐negative rate was 5·9 per cent with the dual‐modality technique, in comparison with 8·6 and 7·4 per cent for dye‐only and tracer‐only techniques respectively^6^. The dual technique, nevertheless, has shortcomings, including radiation exposure to healthcare professionals and patients, issues with radiotracer availability, dependency on availability of nuclear medicine units and allergic reactions to BD.

New techniques have been developed to improve the clinical value of sentinel lymph node biopsy with similar accuracy, but avoiding irradiation and risks of allergy. Novel techniques studied in recent years include those using indocyanine green (ICG) fluorescence, superparamagnetic iron oxide (SPIO) nanoparticles, and contrast‐enhanced ultrasound imaging using microbubbles (CEUS). A systematic review by Ahmed and colleagues^7^ in 2014 reported that these techniques showed clinical potential but had high false‐negative rates. The review was based on pairwise meta‐analysis and was unable to compare relative performance between techniques not previously studied in a direct comparison. The aim of the present network meta‐analysis was to compare these techniques directly and indirectly in terms of detection and false‐negative rates.

## Methods

### Literature search and eligibility criteria

A literature search was performed in electronic databases, including PubMed, Embase, the Cochrane Library, China Knowledge Research Integrated Database, ClinicalTrials.gov and OpenGrey, up to 31 November 2017. The specific concepts used in the search strategy were ‘Breast Neoplasms’, ‘Sentinel Lymph Node Biopsy’, ‘Indocyanine Green’, ‘Superparamagnetic Iron Oxide’ and ‘Contrast‐enhanced Ultrasound’. Medical Subject Headings (MeSH) or Emtree, and free‐text terms were both searched (*Table S1*, supporting information). There were no restrictions on language. All bibliographies listed in relevant review papers and included publications were also checked.

Two reviewers independently searched for eligible studies based on predefined eligibility criteria. Studies that compared the detection performance of at least one of the novel methods (ICG, SPIO and CEUS) with that of traditional methods (BD and/or Tc) were included. Single‐arm studies that assessed only one of the methods, reviews, case reports, animal studies, commentaries and letters to editors were excluded. Discrepancies were discussed between reviewers until consensus was reached.

### Data extraction and quality assessment

The following data were extracted from the included studies: study characteristics (publication year, number of subjects); baseline characteristics (mean age and recruitment method); and outcome events (number of detections and number of false‐negative reports). Relevant data were also extracted for quality assessment.

A network geometry was constructed based on the included studies^8^. Each node represented various techniques, and the size was weighted by the number of subjects from whom data on each technique were derived. The connecting line between two nodes represented the presence of a direct comparison, and the number of studies included determined the thickness of each line. Hence, the thicker the line, the more studies comparing the two methods were included.

The quality of each study was evaluated by two independent investigators, using the Risk Of Bias in Non‐randomized Studies of Interventions (ROBINS‐I) tool^9^. Seven domains (confounding, selection of participants into the study, classification of interventions, deviations from intended interventions, missing data, measurement of outcomes and selection of the reported result) were evaluated to assess the risk of bias. For each of the seven domains in the ROBINS‐I tool, responses to the signalling questions were taken together to arrive at the judgement of low, moderate, serious or critical risk of bias, and judgements within each domain were summarized to provide an overall risk‐of‐bias judgement for the outcome being assessed. Any disagreement in quality assessment was resolved by discussion and consensus.

### Statistical analysis

A network meta‐analysis, comparing the detection methods for the proposed outcomes, was performed using a multivariable meta‐regression model with random effects, adopting a frequentist approach. The model allowed the inclusion of potential co‐variables and accounted for correlations from multiarm trials. Risk ratios (RRs) with 95 per cent confidence intervals were estimated for detection rate and false‐negative rate.

To rank the effect size of all the methods, surface under the cumulative ranking (SUCRA) value, indicating the probability of each method being the best, was used^10^. A higher SUCRA value indicated a higher rank of performance for the method.

To check for the assumption of consistency, the design‐by‐treatment approach was used. This approach tested whether the difference between RRs from direct comparison (either by 2‐arm or multiarm design) and those from network meta‐analysis was statistically significant. The χ^2^ test was used to test the difference throughout the entire network, and *P* < 0·050 indicated the need for further investigation to identify the sources of inconsistency^11^.

The network meta‐analyses were generated using Stata/MP® version 13 with network and network graphs packages^12^, ^13^, ^14^.

## Results

### Study characteristics and network geometry

**Figure 1:**
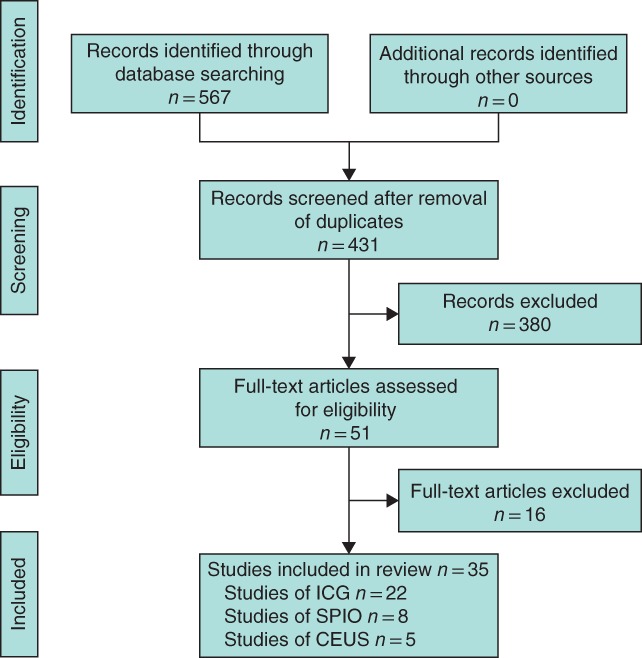
PRISMA flow chart showing selection of studies for review. ICG, indocyanine green; SPIO, superparamagnetic iron oxide; CEUS, contrast‐enhanced ultrasound imaging

Of 567 studies identified from the initial search, 35 cohort studies^15^, ^16^, ^17^, ^18^, ^19^, ^20^, ^21^, ^22^, ^23^, ^24^, ^25^, ^26^, ^27^, ^28^, ^29^, ^30^, ^31^, ^32^, ^33^, ^34^, ^35^, ^36^, ^37^, ^38^, ^39^, ^40^, ^41^, ^42^, ^43^, ^44^, ^45^, ^46^, ^47^, ^48^, ^49^ (4244 subjects) satisfied the inclusion and exclusion criteria, and were included in this meta‐analysis *(Fig*. 1, *Table* 1). Among the studies included, 22^15,16,20–23,26,27,30–32,34,35,38–41,43–45,47,49^ reported on the performance of ICG, eight^17^, ^19^, ^24^, ^25^, ^29^, ^33^, ^37^, ^42^ on SPIO and five^18^, ^28^, ^36^, ^46^, ^48^ on CEUS. The mean age of included subjects was 57 years.

**Table 1:** Baseline characteristics of included studies

Reference	Country	Methods compared	No. of subjects	Mean age (years)	Recruitment method
Abe *et al*.^15^	Japan	BD, ICG	128	52·6	Non‐consecutive
Ballardini *et al*.^16^	Italy	Tc, ICG	134	56	Non‐consecutive
Douek *et al*.^17^	UK, Netherlands	Tc/BD, SPIO	160	n.a.	Non‐consecutive
Esfehani *et al*.^18^	Iran	Tc/BD, CEUS	50	53	Non‐consecutive
Ghilli *et al*.^19^	Italy	Tc, SPIO	193	61	Consecutive
Grischke *et al*.^20^	Germany	Tc, ICG	105	57·5	Non‐consecutive
Guo *et al*.^21^	China	BD, ICG	86	52·3	Consecutive
Hirano *et al*.^22^	Japan	BD, ICG	108	60·5	Non‐consecutive
Hojo T *et al*.^23^	Japan	BD, ICG	131	57·6	Non‐consecutive
Houpeau *et al*.^24^	France	Tc/BD, SPIO	108	58	Non‐consecutive
Karakatsanis *et al*.^25^	Sweden	Tc, SPIO	206	61·7	Non‐consecutive
Mieog *et al*.^26^	USA	BD, Tc, ICG	24	59·5	Consecutive
Murawa *et al*.^27^	Japan	Tc, ICG	20	56	Non‐consecutive
Omoto *et al*.^28^	Japan	BD, Tc, CEUS	20	50·9	Non‐consecutive
Piñero‐Madrona *et al*.^29^	Spain	Tc/BD, SPIO	181	56	Non‐consecutive
Pitsinis *et al*.^30^	UK	BD, ICG	50	48	Consecutive
Polom *et al*.^31^	Poland	Tc, ICG	13	55	Consecutive
Rauch *et al*.^32^	Austria	BD, Tc, ICG	98	61·9	Consecutive
Rubio *et al*.^33^	Spain	Tc, SPIO	118	n.a.	Non‐consecutive
Samorani *et al*.^34^	Japan	Tc, ICG	301	n.a.	Consecutive
Schaafsma *et al*.^35^	Netherlands	Tc, BD, ICG	32	56	Consecutive
Sever *et al*.^36^	UK	BD, Tc, CEUS	54	60·5	Consecutive
Shiozawa *et al*.^37^	Japan	BD, SPIO	30	58·2	Non‐consecutive
Stoffels *et al*.^38^	Germany	Tc, ICG	80	55	Non‐consecutive
Sugie *et al*.^39^	Japan	BD, ICG	99	60	Non‐consecutive
Sugie *et al*.^40^	Japan	Tc, ICG	821	55	Consecutive
Tagaya *et al*.^41^	Japan	BD, ICG	25	n.a.	Consecutive
Thill *et al*.^42^	Germany	Tc, SPIO	150	57·6	Non‐consecutive
Tong *et al*.^43^	China	BD, ICG	96	54·2	Non‐consecutive
van der Vorst *et al*.^44^	Netherlands	Tc, BD, ICG	24	60·5	Consecutive
Verbeek *et al*.^45^	Netherlands, USA	Tc, BD, ICG	95	57	Non‐consecutive
Wang *et al*.^46^	China	BD, CEUS	46	50·5	Consecutive
Wishart *et al*.^47^	UK	Tc, BD, ICG	99	n.a.	Non‐consecutive
Xie *et al*.^48^	China	BD, CEUS	101	n.a.	Consecutive
Yamamoto *et al*.^49^	Japan	BD, ICG	258	57	Consecutive

BD, blue dye alone; ICG, indocyanine green; Tc, technetium‐99m; Tc/BD, combined use of technetium‐99m and blue dye; SPIO, superparamagnetic iron oxide; n.a., not available; CEUS, contrast‐enhanced ultrasound imaging.

**Figure 2:**
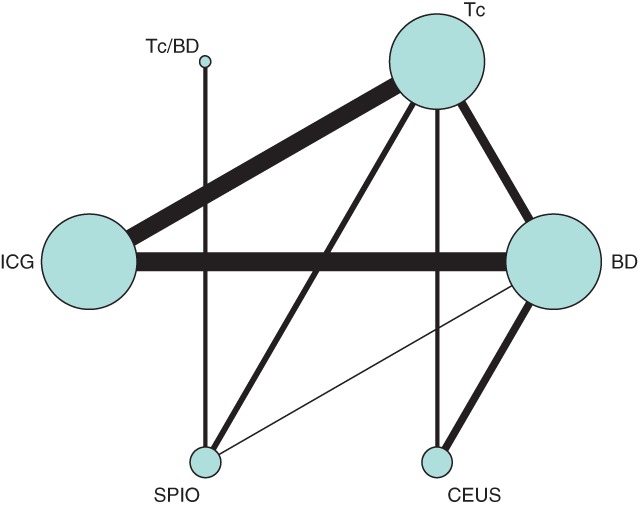
Network geometry of included studies. Tc/BD, combined use of technetium‐99m and blue dye; Tc, technetium‐99m; ICG, indocyanine green; BD, blue dye; SPIO, superparamagnetic iron oxide; CEUS, contrast‐enhanced ultrasound imaging

The network geometry summarized the studies visually (*Fig*. 2). Tc, BD and ICG were the three most reported detection methods. Tc, BD and ICG fluorescence groups included most of the subjects. There were no direct comparisons between ICG, SPIO and CEUS.

Quality evaluation of all the studies demonstrated a low to moderate risk of bias in the seven domains assessed, and 20 of 35 had a moderate risk of bias on overall assessment (*Table S2*, supporting information). Moderate risk of bias was assigned in terms of patient selection to studies where the details of recruitment were not clearly reported.

### Detection rate

A total of 35 studies^15^, ^16^, ^17^, ^18^, ^19^, ^20^, ^21^, ^22^, ^23^, ^24^, ^25^, ^26^, ^27^, ^28^, ^29^, ^30^, ^31^, ^32^, ^33^, ^34^, ^35^, ^36^, ^37^, ^38^, ^39^, ^40^, ^41^, ^42^, ^43^, ^44^, ^45^, ^46^, ^47^, ^48^, ^49^ involving 4244 subjects were included in the analysis of detection rate. Pooled detection rates for each method are shown in *Table* 2. Pooled RRs for Tc (1·09, 95 per cent c.i. 1·04 to 1·15), ICG (1·12, 1·07 to 1·16) and SPIO (1·09, 1·01 to 1·18) showed statistically better performance in detecting the sentinel lymph node, approximately 1·1 times better, than BD alone (*Table* 3). Risk differences (RD) compared with BD were 8·0 (95 per cent c.i. 3·7 to 12·3), 10·5 (6·8 to 14·2) and 6·8 (–0·3 to 14·0) per cent for Tc, ICG and SPIO respectively. RRs for the dual‐modality technique (Tc/BD) and CEUS were 1·09 (0·98 to 1·16) and 1·03 (0·98 to 1·11) respectively compared with BD, but were not statistically significant. RDs were 5·9 (–2·4 to 14·2) and 3·6 (–2·5 to 9·6) per cent respectively.

**Table 2:** Pooled estimates of detection rate and false‐negative rate for each method

	Detection rate (%)	False‐negative rate (%)
ICG	97·9 (96·9, 98·9)	0·6 (–0·3, 1·5)
SPIO	97·4 (96·3, 98·6)	4·0 (1·9, 6·1)
CEUS	92·8 (86·7, 98·8)	10·5 (1·7, 19·4)
Tc	96·5 (95·2, 97·9)	2·6 (0·7, 4·6)
Tc/BD	96·7 (94·3, 99·1)	5·5 (0·9, 10·2)
BD	86·8 (82·7, 91·0)	18·4 (11·9, 24·9)

Values in parentheses are 95 per cent confidence intervals. ICG, indocyanine green; SPIO, superparamagnetic iron oxide; CEUS, contrast‐enhanced ultrasound imaging; Tc, technetium‐99m; Tc/BD, combined use of technetium‐99m and blue dye; BD, blue dye alone.

**Table 3:** Pooled risk ratios for detection and false‐negative rates from network meta‐analysis

Test method	Reference method				
	SPIO	CEUS	Tc	Tc/BD	BD
ICG					
RR for detection	1·03 (0·96, 1·11) 0·67 (0·21, 2·08)	1·08 (1·00, 1·15) 0·16 (0·52, 0·05)	1·03 (0·99, 1·06) 0·64 (0·36, 1·13)	1·05 (0·96, 1·14) 0·56 (0·14, 2·23)	1·12 (1·07, 1·16)* 0·29 (0·16, 0·54)*
RR for FN rate					
SPIO					
RR for detection		1·03 (0·95, 1·12) 0·24 (0·05, 1·08)	0·99 (0·93, 1·06) 0·96 (0·34, 2·75)	1·01 (0·95, 1·07) 0·84 (0·36, 1·93)	1·09 (1·01, 1·18)* 0·45 (0·14, 1·45)
RR for FN rate					
CEUS					
RR for detection			0·96 (0·89, 1·02) 4·00 (1·17, 13·7)*	0·98 (0·90, 1·06) 3·49 (0·67, 18·2)	1·03 (0·98, 1·11) 1·85 (0·68, 6·06)
RR for FN rate					
Tc					
RR for detection				1·00 (0·91, 1·10) 0·87 (0·24, 3·23)	1·09 (1·04, 1·15)* 0·44 (0·20, 0·96)*
RR for FN rate					
Tc/BD					
RR for detection					1·09 (0·98, 1·16)
RR for FN rate					0·57 (0·13, 2·51)

Values in parentheses are 95 per cent confidence intervals. Risk ratios (RRs) are shown for test *versus* reference technique. SPIO, superparamagnetic iron oxide; CEUS, contrast‐enhanced ultrasound imaging; Tc, technetium‐99m; Tc/BD, combined use of technetium‐99m and blue dye; BD, blue dye alone; ICG, indocyanine green; FN, false‐negative. *P < 0·050 (multivariable metaregression model with random effects).

ICG had a relatively better detection performance than CEUS (RR 1·08, 1·00 to 1·15), but not SPIO (RR 1·03, 0·96 to 1·11). Absolute RDs were 3·7 (–3·4 to 10·7) per cent for ICG over SPIO, and 7·0 (0·4 to 13·5) per cent for ICG over CEUS.

The ranking for performance in terms of detection rate, using SUCRA values, is shown in *Table* 4. The rank from highest to lowest was ICG, SPIO, Tc, Tc/BD, CEUS and BD. Of note, Tc/BD had almost the same SUCRA as Tc (60·9 and 61·3 per cent respectively).

**Table 4:** Summary of SUCRA values and mean ranks by detection and false‐negative rates

	Detection rate		False‐negative rate	
	SUCRA (%)	Rank	SUCRA (%)	Rank
ICG	85·7	1	90·7	1
SPIO	63·8	2	65·6	2
Tc	61·3	3	64·1	3
Tc/BD	60·9	4	50·4	4
CEUS	23·0	5	4·5	6
BD	5·3	6	24·6	5

SUCRA, surface under the cumulative ranking; ICG, indocyanine green; SPIO, superparamagnetic iron oxide; Tc, technetium‐99m; Tc/BD, combined use of technetium‐99m and blue dye; CEUS, contrast‐enhanced ultrasound imaging; BD, blue dye alone.

### False‐negative rate

Some 25 studies^15^, ^17^, ^19^, ^20^, ^21^, ^22^, ^23^, ^24^, ^25^, ^27^, ^28^, ^29^, ^32^, ^33^, ^34^, ^35^, ^37^, ^38^, ^39^, ^40^, ^41^, ^42^, ^43^, ^45^, ^48^ were included in the analysis of false‐negative rate. Pooled false‐negative rates for each technique are shown in *Table* 2. The lowest RR was seen in the ICG group (0·29, 95 per cent c.i. 0·16 to 0·54) followed by Tc (RR 0·44, 0·20 to 0·96) and SPIO (RR 0·45, 0·14 to 1·45), using BD as the reference group (*Table* 3). Similarly, RDs showed a reduction of 18·1 (95 per cent c.i. –24·2 to –12·0) per cent for ICG *versus* BD, 16·1 (–22·8 to –9·4) per cent for Tc *versus* BD and 15·5 (–23·2 to 7·9) per cent for SPIO versus BD. The risk of a false‐negative result with CEUS was twice that for BD (RR 1·85, 0·68 to 6·06).

The SUCRA results for false‐negative rate showed a similar pattern to those for detection rate; CEUS and BD had a low SUCRA rank of 6 and 5 respectively, which translates to higher false‐negative rate compared with other modalities (*Table* 4).

### Network consistency

Forest plots comparing pooled estimates from both direct comparison and network meta‐analysis were reported for detection rate (*Fig. S1*, supporting information) and false‐negative rate (*Fig. S2*, supporting information). On visual check, greater consistency existed among pooled estimates from all two‐arm studies, all multiarm studies and network meta‐analysis. The consistency assumption was examined statistically using the χ^2^ test, which yielded *P* = 0·316 for detection rate and *P* = 0·948 for false‐negative rate, indicating no evidence for the potential violation of assumption. There was, therefore, no statistically significant evidence of inconsistency.

## Discussion

In this review, the ICG fluorescence technique ranked first in terms of detection, as well as having the lowest false‐negative rate of the techniques compared. The ranking of other techniques in descending order was SPIO, Tc, dual modality (Tc/BD), CEUS and BD in terms of detection rate.

The use of ICG fluorescence for sentinel lymph node biopsy in patients with breast cancer was first described in 1999 by Motomura and colleagues^50^, who reported an identification rate of 73·8 per cent. The main advantages of this method are accurate placement of the skin incision as subcutaneous lymphatic channels can be identified on the skin before operation, and accuracy is improved with the use of fluorescence as there is high sensitivity with illumination even if the nodes do not stain green. On the contrary, problems and technical difficulties associated with this technique include difficulty with ICG fluorescence detection for lymph nodes more than 1 cm below the skin, need for operating theatre lights to be turned off during fluorescence navigation, and diffusion of ICG into subcutaneous tissue if the procedure takes more than 30 min, reducing the sensitivity of fluorescence detection. In a 2016 meta‐analysis^51^, the pooled detection rate for this technique was 0·98 (95 per cent c.i. 0·96 to 0·99) with a false‐negative rate of 8 per cent. The main limitation of the meta‐analysis was that only six studies were included in the final analysis. The present meta‐analysis included 22 studies that used ICG. This technique achieved a significant 12 per cent improvement in terms of detection rate and a 71 per cent lower false‐negative rate compared with BD.

SPIO nanoparticles (Sienna+®; Sysmex Europe, Hamburg, Germany) have gained interest since the first description of their use in 2013^52^. SPIO nanoparticles stay in the tissue for a prolonged period and can be detected with a magnetometer^25^. This characteristic has made it feasible for SPIO to be injected in the outpatient clinic 3–15 days before surgery, with a success rate comparable to that of Tc/BD^53^. A meta‐analysis^54^ of seven randomized trials in 2016 showed that the SPIO method was not inferior to the standard technique in identification rate (97·1 *versus* 96·8 per cent), total lymph nodes retrieved (1·9 *versus* 1·8 nodes per patient) or false‐negative rate (8·4 *versus* 10·9 per cent). Using both direct and indirect comparisons, the present analysis found that SPIO performed significantly better than BD in terms of detection rate. Although the RR compared with BD for false‐negative rate was not statistically significant, a 55 per cent decrease on the point estimate suggested a promising direction for the use of SPIO in sentinel lymph node biopsy.

CEUS with microbubbles, based on the use of dispersion with sulphur hexafluoride gas injected intradermally around the areola, for sentinel node biopsy was first reported in 2013^55^. In a subsequent meta‐analysis of five studies^56^, this technique identified sentinel lymph nodes in 9·3–55·2 per cent of patients, with a sensitivity of 61–89 per cent and a false‐negative rate of 6·6–39 per cent. No comparative study of CEUS against the dual‐modality technique exists to validate this method. In the present analysis, there was no statistical difference between CEUS and BD in terms of detection rate, but the risk of a false‐negative result was four times that for the Tc method (RR 4·00, 95 per cent c.i. 1·17 to 13·70). The high false‐negative rate might be attributable to inconsistent identification of sentinel lymph nodes owing to interobserver variability, even when the same ultrasound equipment is used. The introduction of super‐resolution imaging, ultrafast ultrasound imaging and improved microbubble transit may improve the visualization of lymphatics and sentinel lymph nodes with this technique^55^.

It is acknowledged that this review has inherent limitations as evaluation of the performance of each technique was limited to detection and false‐negative rates. From a clinical standpoint, the strength of a particular modality is also dependent on other factors such as ease of use and side‐effects. It would be of interest to investigate other details pertaining to the use of these techniques, such as dose and site of injection, to see how these affect performance. Limitations in terms of statistical analysis related to the lack of RCTs, although the risk of bias was generally low to moderate among the included studies.

The strength of the present review is the use of network meta‐analysis to allow direct and indirect comparisons between techniques for sentinel lymph node identification and false‐negative rates. Previous systematic reviews evaluated various techniques qualitatively, rather than quantitatively, making conclusions less convincing. By synthesizing direct and indirect evidence using network meta‐analysis, this study was able to rank the detection and false‐negative rates across all techniques quantitatively.

Both ICG and SPIO consistently performed better than BD alone in this review. Based on this finding, these methods could provide a reliable alternative in centres where BD is used as a single modality, as the use of either technique would result in better detection and false‐negative rates. Although it is interesting to note that these two novel methods had similar performance to radioactive colloid in terms of detection as well as false‐negative rates, technical, logistical and economic factors should be taken into consideration. Studies comparing novel methods with standard dual‐modality techniques are still needed, in the hope of demonstrating superior performance.

In this network meta‐analysis, Tc, ICG and SPIO demonstrated superior performance over BD in terms of detection and false‐negative rate. Future cost‐effectiveness analyses will be crucial to determine whether the novel techniques are sustainable in the long run.

## Disclosure

The authors declare no conflict of interest.

## Supporting information


**Table S1.** Search strategies
**Table S2.** Risk of bias assessment
**Fig. S1.** Forest plot of all direct comparison on detection rate together with pooled estimates from network meta‐analysis
**Fig. S2.** Forest plot of all direct comparison on false negative rate together with pooled estimates from network meta‐analysis
