# Supplementary material for: Network meta‐analysis of novel and conventional sentinel lymph node biopsy techniques in breast cancer
Source: BJS Open. 2019 Mar 25;3(4):445–52. doi: 10.1002/bjs5.50157 (PMC6677105; doi:10.1002/bjs5.50157)
Supplement: Supplementary file 1 — Table S1. Search strategies Table S2. Risk of bias assessment Fig. S1. Forest plot of all direct comparison on detection rate together with pooled estimates from network meta‐analysis Fig. S2. Forest plot of all direct comparison on false negative rate together with pooled estimates from network meta‐analysis [file BJS5-3-445-s001.docx]

**BJS5_50157**

**Network meta-analysis of novel and conventional sentinel lymph node biopsy techniques in breast cancer**

C. W. Mok, S.-M. Tan, Q. Zheng and L. Shi

Table S1 Search strategies

| **MEDLINE/Pubmed:** |
| --- |
| #1 (((((("Breast Neoplasms"[Mesh]) OR Breast Neoplasm*) OR Breast cancer*) OR Breast carcin*) OR Breast metasta*) OR Breast malig*) OR Breast tumo* |
| #2 ((("Sentinel Lymph Node Biopsy"[Mesh]) OR Sentinel Lymph Node*)) OR (SLN OR SLNB) |
| #3 (((("Indocyanine Green"[Mesh]) OR Indocyanine Green) OR fluorescence)) OR ICG |
| #4 ((((superparamagnetic iron oxide) OR (SPIO OR SPIOS OR SPION OR SPIONS))) OR magnetic tracer*) OR magnetic technique* |
| #5 (((microbubbles[MeSH Terms]) OR (microbubbles OR microbubble))) AND (((Ultrasonography[MeSH Terms]) OR contrast-enhanced ultrasound) OR CEU OR CEUS) |
| #6 #1 AND #2 AND (#3 OR #4 OR #5) |
|  |
| **Embase:** |
| #1 'breast cancer'/exp OR 'breast cancer' OR 'breast neoplasm$' OR 'breast cancer$' OR 'breast carcin*' OR 'breast metasta*' OR 'breast malig*' OR 'breast tumo*' |
| #2 'sentinel lymph node biopsy'/exp OR 'sentinel lymph node biopsy' OR 'sentinel lymph node$' OR sln OR slnb |
| #3 'indocyanine green'/exp OR 'indocyanine green' OR 'indocyanine green angiography'/exp OR 'indocyanine green angiography' OR ﬂuorescence OR icg |
| #4 'superparamagnetic iron oxide nanoparticle'/exp OR 'superparamagnetic iron oxide nanoparticle' OR 'superparamagnetic iron oxide'/exp OR 'superparamagnetic iron oxide' OR spio OR spios OR 'spion' OR 'spion'/exp OR spion OR spions OR 'magnetic tracer$' OR 'magnetic technique$' |
| #5 'microbubble'/exp OR 'microbubble' OR 'microbubbles'/exp OR 'microbubbles' AND ('ultrasound'/exp OR 'ultrasound' OR 'contrast enhanced' AND ('ultrasound' OR 'ultrasound'/exp OR ultrasound) OR ceu OR ceus) |
| #5 #1 AND #2 AND (#3 OR #4 OR 5) |
|  |
| **Cochrane Library:** |
| #1 MeSH descriptor: [Sentinel Lymph Node Biopsy] explode all trees |
| #2 entinel Lymph Node* or SLN or SLNB |
| #3 MeSH descriptor: [Breast Neoplasms] explode all trees |
| #4 Breast Neoplasm* or Breast cancer* or Breast carcin* or Breast metasta* or Breast malig* or Breast tumo* |
| #5 MeSH descriptor: [Indocyanine Green] explode all trees |
| #6 Indocyanine Green or ﬂuorescence or ICG |
| #7 superparamagnetic iron oxide or SPIO or SPIOS or SPION or SPIONS or magnetic tracer* or magnetic technique* |
| #8 MeSH descriptor: [Microbubbles] explode all trees |
| #9 microbubbles or microbubble |
| #10 MeSH descriptor: [Ultrasonography] explode all trees |
| #11 contrast-enhanced ultrasound or CEU or CEUS |
| #12 (#1 or #2) and (#3 or #4) and (#5 or #6 or #7 or ((#8 or #9) and (#10 or #11))) |

Table S2 Risk of bias assessment

| **Study** | **Bias due to confounding** | **Bias in selection of participants into the study** | **Bias in classification of interventions** | **Bias due to deviations from intended interventions** | **Bias due to missing data** | **Bias in measurement of outcomes** | **Bias in selection of the reported result** | **Overall** |
| --- | --- | --- | --- | --- | --- | --- | --- | --- |
| Abe H, 2011[^15^](#_ENREF_15) | Low | Moderate | Low | Low | Low | Low | Low | Moderate |
| Ballardini B, 2013[^16^](#_ENREF_16) | Low | Moderate | Low | Low | Low | Low | Low | Moderate |
| Douek M, 2014[^17^](#_ENREF_17) | Low | Moderate | Low | Low | Low | Low | Low | Moderate |
| Esfehani MH, 2015[^18^](#_ENREF_18) | Low | Moderate | Low | Low | Low | Low | Low | Moderate |
| Ghilli M, 2015[^19^](#_ENREF_19) | Low | Low | Low | Low | Low | Low | Low | Low |
| Grischke EM, 2015[^20^](#_ENREF_20) | Low | Moderate | Low | Low | Low | Low | Low | Moderate |
| Guo W, 2014[^21^](#_ENREF_21) | Low | Low | Low | Low | Low | Low | Low | Low |
| Hirano A, 2012[^22^](#_ENREF_22) | Low | Moderate | Low | Low | Low | Low | Low | Moderate |
| Hojo T, 2010[^23^](#_ENREF_23) | Low | Moderate | Low | Low | Low | Low | Low | Moderate |
| Houpeau JL, 2016[^24^](#_ENREF_24) | Low | Moderate | Low | Low | Low | Low | Low | Moderate |
| Karakatsanis A, 2016[^25^](#_ENREF_25) | Low | Moderate | Low | Low | Low | Low | Low | Moderate |
| Mieog JSD, 2011[^26^](#_ENREF_26) | Low | Low | Low | Low | Low | Low | Low | Low |
| Murawa D, 2009[^27^](#_ENREF_27) | Low | Moderate | Low | Low | Low | Low | Low | Moderate |
| Omoto K, 2009[^28^](#_ENREF_28) | Low | Moderate | Low | Low | Low | Low | Low | Moderate |
| Piñero-Madrona A, 2015[^29^](#_ENREF_29) | Low | Moderate | Low | Low | Low | Low | Low | Moderate |
| Pitsinis V, 2015[^30^](#_ENREF_30) | Low | Low | Low | Low | Low | Low | Low | Low |
| Polom K, 2011[^31^](#_ENREF_31) | Low | Low | Low | Low | Low | Low | Low | Low |
| Rauch, 2017[^32^](#_ENREF_32) | Low | Low | Low | Low | Low | Low | Low | Low |
| Rubio IT, 2015[^33^](#_ENREF_33) | Low | Moderate | Low | Low | Low | Low | Low | Moderate |
| Samorani D, 2015[^34^](#_ENREF_34) | Low | Low | Low | Low | Low | Low | Low | Low |
| Schaafsma BE, 2013[^35^](#_ENREF_35) | Low | Low | Low | Low | Low | Low | Low | Low |
| Sever A, 2009[^36^](#_ENREF_36) | Low | Low | Low | Low | Low | Low | Low | Low |
| Shiozawa M, 2013[^37^](#_ENREF_37) | Low | Moderate | Low | Low | Low | Low | Low | Moderate |
| Stoffels I, 2015[^38^](#_ENREF_38) | Low | Moderate | Low | Low | Low | Low | Low | Moderate |
| Sugie T, 2013[^39^](#_ENREF_40) | Low | Moderate | Low | Low | Low | Low | Low | Moderate |
| Sugie T, 2016[^40^](#_ENREF_39) | Low | Low | Low | Low | Low | Low | Low | Low |
| Tagaya N, 2008[^41^](#_ENREF_41) | Low | Low | Low | Low | Low | Low | Low | Low |
| Thill M, 2014[^42^](#_ENREF_42) | Low | Moderate | Low | Low | Low | Low | Low | Moderate |
| Tong M, 2014[^43^](#_ENREF_43) | Low | Moderate | Low | Low | Low | Low | Low | Moderate |
| van der Vorst JR, 2012[^44^](#_ENREF_44) | Low | Low | Low | Low | Low | Low | Low | Low |
| Verbeek FPR, 2014[^45^](#_ENREF_45) | Low | Moderate | Low | Low | Low | Low | Low | Moderate |
| Wang, 2017[^46^](#_ENREF_46) | Low | Low | Low | Low | Low | Low | Low | Low |
| Wishart GC, 2012[^47^](#_ENREF_47) | Low | Moderate | Low | Low | Low | Low | Low | Moderate |
| Xie F, 2015[^48^](#_ENREF_48) | Low | Low | Low | Low | Low | Low | Low | Low |
| Yamamoto S, 2013[^49^](#_ENREF_49) | Low | Low | Low | Low | Low | Low | Low | Low |

**Fig. S1 Forest plot of all direct comparison on detection rate together with pooled estimates from network meta-analysis**

BD: Blue dye; CEUS: Contrast-enhanced ultrasound; ICG: Indocyanine green; SPIO: Superparamagnetic iron oxide; Tc: Technetium-99; Tc/BD: Combined used of Technetium-99 and blue dye; NMA: Network meta-analysis

Fig. S2 Forest plot of all direct comparison on false negative rate together with pooled estimates from network meta-analysis

Blue dye; CEUS: Contrast-enhanced ultrasound; ICG: Indocyanine green; SPIO: Superparamagnetic iron oxide; Tc: Technetium-99; Tc/BD: Combined used of Technetium-99 and blue dye; NMA: Network meta-analysis
